# K-line tilt as a novel potential risk factor for cervical Modic change: a retrospective study

**DOI:** 10.1186/s13018-023-03780-y

**Published:** 2023-04-11

**Authors:** Qingsong Zhou, Wei Deng, Shengtao Wang, Jieyong Cai, Junfei Feng, Qian Chen, Yong Yin

**Affiliations:** 1 Department of Orthopedics, Pidu District People’s Hospital, The Third Affiliated Hospital of Chengdu Medical College, Chengdu, 611730 China; 2 Department of Orthopedics and Laboratory of Biological Tissue Engineering and Digital Medicine, Affiliated Hospital of North Sichuan Medical College, Nanchong, 637000 China

**Keywords:** Modic changes of cervical spine, K-line tilt, Sagittal parameters of cervical spine

## Abstract

**Background:**

Cervical sagittal parameters are important parameters that reflect the mechanical stress in the sagittal plane of the cervical spine and are an important basis for predicting the clinical status and prognosis of patients. Although it has been confirmed that there is a significant correlation between cervical Modic changes and some sagittal parameters. However, as a newly discovered sagittal parameter, there is no report on the relationship between the K-line tilt and the Modic changes of cervical spine.

**Methods:**

A retrospective analysis was performed for 240 patients who underwent cervical magnetic resonance imaging scan for neck and shoulder pain. Among them, 120 patients with Modic changes, namely the MC(+) group, were evenly divided into three subgroups of 40 patients in each group according to different subtypes, namely MCI subgroup, MCII subgroup and MCIII subgroup. One hundred twenty patients without Modic changes were included in MC(−) group. We measured and compared the sagittal parameters of cervical spine among different groups, including K-line tilt, C2–C7 sagittal axial vertical distance (C2–C7 SVA), T1 slope and C2–7 lordosis. Logistic regression was used to analyse the risk factors of cervical Modic changes.

**Results:**

The K-line tilt and C2–7 lordosis were significantly different between MC(+) group and MC(−) group (*P* < 0.05). The K-line tilt greater than 6.72° is a risk factor for Modic changes in cervical spine (*P* < 0.05). At the same time, the receiver operating characteristic curve showed that this change had moderate diagnostic value when the area under the curve was 0.77.

**Conclusion:**

This study shows that the K-line tilt greater than 6.72° is a potential risk factor for Modic changes in cervical spine. When the K-line tilt is greater than 6.72°, we should be alert to the occurrence of Modic changes.

*Trial registration number*: 2022ER023-1.

## Background

Neck pain is one of the important diseases affecting the quality of life of middle-aged and elderly people, which has brought great social and economic burden to the world and has become a global public health problem [1, 2]. Therefore, it is urgent to increase the understanding of neck pain. The causes of neck pain are varied, however, Modic changes in the cervical spine are thought to be a factor in an important relationship with neck and shoulder pain [3, 4].

Modic changes refer to abnormal signal changes of vertebral endplate and bone under endplate in MRI examination of spine [5]. According to the signal changes of endplate on MRI, it can be divided into three types [6, 7]: Modic I changes: low signal on T1WI and high signal on T2WI; Modic II changes: hyperintense on T1WI, isointense or slightly hyperintense on T2WI; Modic III changes: both T1WI and T2WI showed low signal intensity. Previous studies have shown that spinal Modic changes are closely related to the mechanical state of the spine [8, 9]. Disruption of the intervertebral biomechanical balance and increased abnormal stress between adjacent vertebral bodies can lead to spondylolisthesis and vertebral endplate and sub-endplate bone injury, and different periods of injury and repair are correspondingly manifested as different types of Modic changes.

The sagittal parameters of cervical spine are important parameters to reflect the mechanics of cervical spine and the mechanical stress in the sagittal plane of vertebral body and are important basis for evaluating the clinical status of patients and predicting the prognosis of diseases [10]. Previous studies on the relationship between sagittal parameters of cervical spine and Modic changes have found that the larger T1 slope and the smaller and C2–7 lordosis are closely related to the occurrence and development of cervical spine Modic changes [11–13].

K-line is a line connecting C2 and C7 spinal canal centres [14], and the K-line tilt is the included angle between K-line and vertical line, which is a new sagittal parameter of cervical vertebra proposed by scholars [15]. Like traditional parameters, the K-line tilt has important reference value in clinical decision-making and evaluation of disease prognosis [16]. For example, when the K-line tilt is greater than 23.75°, the clinical prognosis of patients undergoing anterior cervical discectomy and decompression and fusion (ACDF) with two adjacent segments is poor [17]. However, there is no research report on the relationship between the K-line tilt and cervical Modic changes. The purpose of this study is to explore the relationship between K-line tilt and cervical Modic changes.

## Methods

### Participants

A total of 240 patients who were hospitalized in our hospital between January 2016 and July 2019 and met the inclusion criteria were randomly selected. Grouping was performed according to changes in MRI endplate signals. In the MC(−) group, T1WI and T2WI showed normal signals. In the MCI group, the signal was low on T1WI and high on T2WI. In group MCII, T1WI showed high signal, T2WI showed isosignal or mild high signal. Both T1WI and T2WI in group MCIII showed low signal. Among them, 120 cases were divided into MC(+) group with 40 cases in each group, which were divided into MCI group, MCII group and MCIII group according to different MRI stages. One hundred twenty patients without Modic changes were included in the control group MC(−). Inclusion criteria: ① continuous neck pain for more than 6 months; ② There are no other structural changes except single segment Modic changes in cervical spine. Exclusion criteria: ① combined with spinal tumour, spinal infection, rheumatoid arthritis and other systemic diseases; ② Previous history of cervical trauma or operation. ③Patients with incomplete imaging data or difficult measurement. The research was approved by the Ethics Review Committee of our hospital. (2022ER023-1).

### Examination procedures

**Fig. 1:**
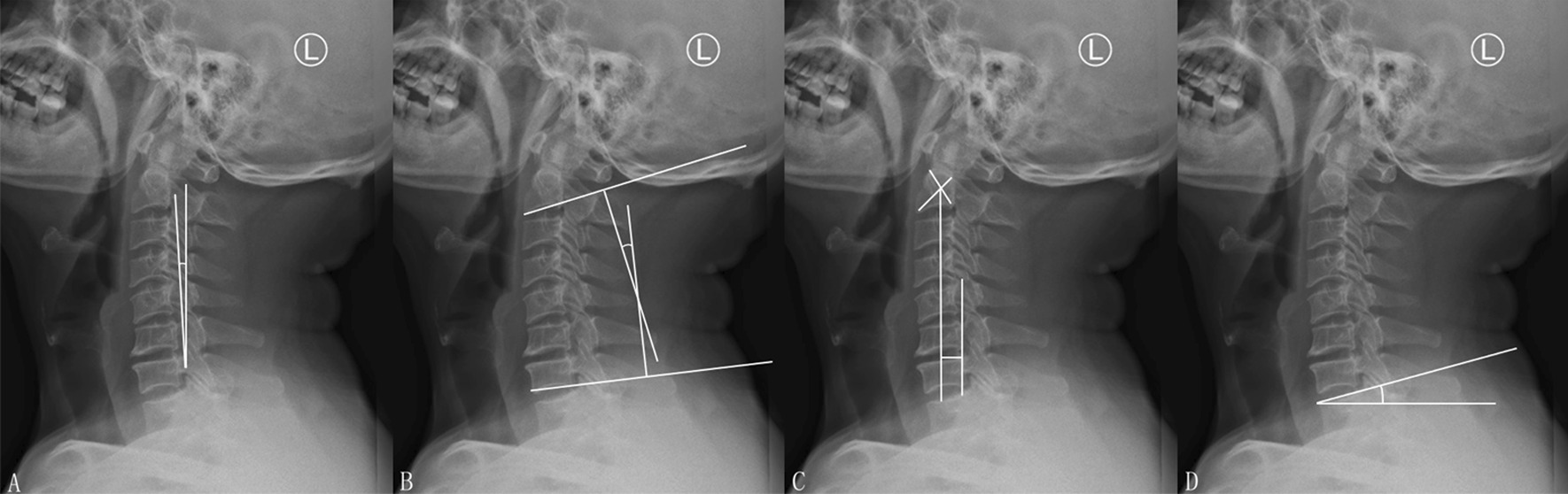
**A** K-line tilt, **B** C2–7 lordosis, **C** C_2_–C_7_ SVA, **D** T1 slope

All Modic changes were judged by cervical MRI, and the sagittal parameters of cervical spine were measured by PACS system on cervical X-ray images. The parameters are defined as follows:

① K-line tilt: the included angle between K-line and vertical line;
② C2–7 lordosis: the angle between C2 lower endplate and C7 lower endplate;
③ C2–C7 SVA: the distance between the vertical line at the centre of C2 vertebral body and the posterior upper edge of C7 vertebral body;
④ T1 slope: the included angle between the upper end plate of T1 and the horizontal line (Fig. 1).

## Data analysis

SPSS 22. 0 software was used for statistical analysis of the data. Age and cervical sagittal position parameters were continuous numerical variables consistent with normal distribution, expressed as means ± standard deviations, and independent sample T test was used. Chi-square test was used for sex, smoking and drinking. Analysis of variance was used among subgroups of MC(+) group. Multivariate Logistic regression was used to analyse the independent risk factors of Modic changes. Construct the ROC curve of the K-line tilt. All test levels α are set to 0.05, where *P* < 0.05 indicates that the difference is significant.

## Results

### Main characteristics of patients

There were no significant difference in age, sex, smoking or alcohol consumption between MC(+) group and MC(−) group. (Table 1).

**Table 1:** Difference analysis of general data

Characteristics	MC(-) (n = 120)	MC (+) (n = 40/n = 40/n = 40)					$\chi^{2}$ or t	*P*
		MCI	MCII	MCIII	F or $\chi^{2}$	*P*		
Age	63.45 ± 12.33	65.5 ± 14.07	65.88 ± 13.37	67.85 ± 13.48	0.34	0.71	1.77	0.08
Gender (male/female)	51/69	18/22	20/20	21/19	0.47	0.79	1.07	0.30
Smoking history (no/yes)	96/24	28/12	32/8	32/8	1.49	0.48	0.39	0.53
Drinking history (no/yes)	96/24	33/7	36/4	36/4	1.37	0.50	2.48	0.12
Position of MC								
C3–C4		7	9	6				
C4–C5		13	12	11				
C5–C6		11	10	12				
C6–C7		9	9	11				

#### Sagittal parameter difference analysis

K-line tilt and C2–7 lordosis are significantly different between MC(+) and MC(−). There were no significant difference between MC(+) and MC(−) in T1 slope and C2-C7 SVA (Table 2).

**Table 2:** Analysis of differences in sagittal parameters

Characteristics	MC(-) (n = 120)	MC ( +) (n = 40/n = 40/n = 40)					$\chi^{2}$ or t	*P*
		MCI	MCII	MCIII	For $\chi^{2}$	*P*		
K-line tilt	5.78 ± 0.92	6.68 ± 0.82	6.49 ± 0.92	6.59 ± 0.73	0.54	0.59	7.20	0.00
T1 slope	23.69 ± 3.95	24.14 ± 2.58	24.33 ± 2.44	24.84 ± 2.42	0.85	0.43	1.75	0.08
C_2_-C_7_ SVA	2.43 ± 0.92	2.56 ± 1.00	2.55 ± 1.05	2.55 ± 1.02	0.00	1.00	1.00	0.32
C2–7 lordosis	11.93 ± 2.64	21.58 ± 4.41	21.16 ± 3.77	20.38 ± 2.76	1.07	0.35	21.91	0.00

#### Multivariate Logistic regression analysis, ROC curve of K-line inclination angle

**Fig. 2:**
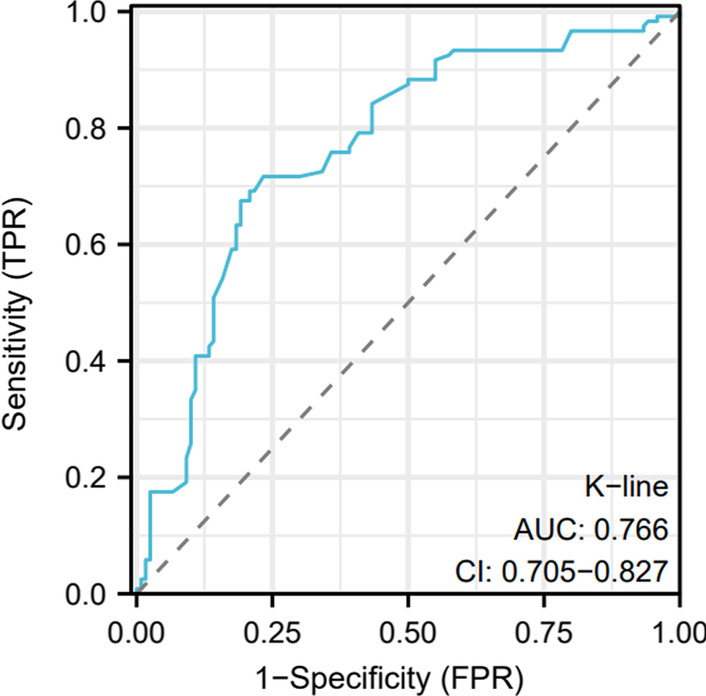
ROC of K-line inclination angle

Age, C2–7 lordosis and K-line tilt are independent risk factors for cervical Modic changes (*p* < 0.05) (Table 3). ROC curve showed that the threshold of K-line tilt on cervical Modic changes was 6.33, and the area under the curve (AUC) was 0.77(*P* < 0.05) (Fig. 2).

**Table 3:** Multivariate logistic regression analysis

	B	S.E	Wald	df	p	Exp(B)	95%CI
K-line tilt	0.94	0.31	9.28	1	0.00	2.56	1.40–4.68
C2–7 lordosis	1.00	0.17	35.41	1	0.00	2.71	1.95–3.77
Age	0.05	0.03	2.90	1	0.09	1.06	1.00–1.12

## Discussion

In this study, we discussed the correlation between Modic changes of cervical spine and sagittal parameters of cervical spine. The results showed that age, C2–7 lordosis and K-line tilt (greater than 6.72°) were independent risk factors for cervical spinal Modic changes.

In recent years, the sagittal parameters of cervical spine are the focus of domestic and foreign scholars, which are important indexes to evaluate the sagittal balance of cervical spine and play an important role in clinical work [18]. In previous studies on the relationship between sagittal parameters of cervical spine and cervical spine Modic changes, it was found that too small C2–7 lordosis was an independent risk factor for predicting cervical spine Modic changes [11], which was consistent with the results of our study. Compared with previous studies, we have added a new sagittal parameter, K-line tilt. The K-line tilt reflects the curvature of cervical vertebra well and plays an important role in maintaining the physiological curve of cervical vertebra and the balance of human body. Its biomechanical function is to increase the ability of cervical vertebra to resist pressure load and cushion the shock to the brain. In addition, compared with other sagittal parameters of cervical spine, the K-line tilt obtained by connecting C2 and C7 spinal canal centres is less disturbed by the image of shoulder and chest, and the angle is more intuitive than the length in the measurement process, without considering the influence of proportion.

The stability of cervical spine can be described by dividing the cervical spine into three main columns, namely, a front column and two rear columns. The anterior column is composed of vertebral body and intervertebral disc, and the posterior column is composed of articular process and joint. The cervical spine maintains a natural forward curvature. This curvature makes the cervical spine mainly distribute the load of the head to the posterior column, which bears about 64% of the load, while the anterior column bears 36% of the load. [19, 20] However, when the K-line tilt becomes larger, the curvature of the cervical spine becomes smaller, so that part of the load is transferred from the posterior column to the anterior column, which increases the mechanical load of the adjacent segments of the cervical vertebral body. The cone endplate is particularly sensitive to mechanical load, and excessive load will lead to bending deformation of cartilage endplate, bone endplate and trabecular meshwork under the plate. At the same time, some studies also showed that excessive compressive force can cause structural damage of vertebral endplate and cancellous bone, and greater load will lead to more microfractures or other forms of injury [21, 22]. This series of changes will promote the occurrence of Modic changes in cervical spine.

In addition, this study found that advanced age is a risk factor for the development of Modic changes in the cervical spine. Wang et al. studied the anatomy of the spinal endplate in 136 male cadavers, showing that age plays an important role in the pathogenesis of endplate lesions, and there is a correlation between age and a variety of endplate lesions [23]. The results of Li et al. also found that the occurrence of Modic changes in the cervical or lumbar spine is age-related. With the increase in natural age, the incidence of endplate damage or calcification increases [24]. In terms of age, atsumoto and other researchers believe that cervical spine Modic changes are more likely to occur in people over 40 years old, and patients older than 40 years have a higher incidence of cervical spine Modic changes [25]. Therefore, for middle-aged and elderly people with large inclination of K-line indicated by clinical X-ray screening, the occurrence of cervical Modic changes should be vigilant, and targeted preventive measures should be implemented early, such as changing bad living habits, to prevent the further development of cervical Modic changes and spinal degeneration. So as to avoid a series of adverse effects caused by cervical Modic changes.

Modic changes were first seen in lumbar spine, but the cervical spine of human body has greater mobility and less load than lumbar spine, so the Modic changes are not exactly the same in cervical spine and lumbar spine. The cervical vertebra, as the segment with the largest mobility of the spine, bears the axial load of the head. Modic changes of cervical spine may lead to neck pain, reduce the curative effect of non-surgical treatment of cervical spine, prolong the time of bone grafting and fusion after ACDF of corresponding cervical spine, and increase the incidence of postoperative axial symptoms [4, 26]. In addition, avascular intervertebral disc tissue provides nutrition through the diffusion of vertebral endplate, and the destruction of vascular structure of endplate by Modic changes will affect the metabolic pathway between vertebral body and intervertebral disc, which may mean that the occurrence and development of Modic changes may aggravate the degeneration of cervical intervertebral disc. At the same time, the degeneration of cervical intervertebral disc can lead to the damage of the normal biomechanical environment between vertebral bodies, the increase in stress between adjacent vertebral bodies, and the bone damage under the vertebral endplate. At present, the mechanism of cervical Modic changes is still unclear. The existing research can only show that cervical Modic changes are closely related to cervical disc degeneration, but the specific causal relationship between them needs further study [27]. Therefore, it is very important for clinical diagnosis, early intervention plan and accelerated functional recovery to increase the understanding of cervical Modic changes [28].

This study has some limitations. First of all, our sample size is small, so although patients are randomly selected, the results are not universal. Secondly, this study is retrospective, so there may be some unintended biases, such as selection bias and information bias. Therefore, in the future research, we hope to further enhance and test the value of this paper through prospective large sample research.

## Conclusion

This study confirmed that the K-line tilt greater than 6.72 is a potential risk factor for cervical spinal Modic changes. When the K-line tilt is greater than 6.72°, we should be alert to the occurrence of cervical Modic changes.
